# “Why can’t we speak in Latvian sometimes?”: learner agency in the language classroom in Latvian diaspora schools

**DOI:** 10.3389/fpsyg.2025.1670007

**Published:** 2026-01-30

**Authors:** Solvita Burr

**Affiliations:** Latvian Language Institute, University of Latvia, Riga, Latvia

**Keywords:** heritage language acquisition, heritage language learner, Latvian-language classroom in Latvian diaspora schools, learner agency, literacy, the Latvian language

## Abstract

The Latvian language is the state language of Latvia. There are about 1.5 million native speakers of Latvian. Of these, 1.38 million live in Latvia, while the rest live across the world. Latvian as a heritage language, or LAT-HL, is taught in about 100 Latvian diaspora weekend schools—hosted by Latvian communities around the world. Although, there is a noticeable increase of research in LAT-HL, they focus on family language policy and practices and the socio-emotional dimension of LAT-HL maintenance in diaspora or, conversely, reimmigration. Considering this gap in LAT-HL research, this article focuses specifically on the perspective of LAT-HL learners and their agency in the Latvian-language lessons. It is based on data obtained from fieldwork at four Latvian diaspora schools (one school each in Europe, the US, Canada, and Australia) in 2025: student electronic questionnaires, interviews with students and Latvian-language teachers, texts created by students, and Latvian-language lesson observation checklists. As a result of data analysis, two thematic groups suggesting student agency were created: language management in the LAT-HL lessons and student involvement/engagement in the creation of the learning process and space. In both cases, the manifestation of learner agency is closely related to (1) the teaching methodology chosen by the teacher, which students accepted or not; (2) the roles of the teacher and students in the classroom; (3) the freedom of choice of students, which was suggested by the teacher or circumstances.

## 1 Introduction

The Latvian language is the state language of Latvia, one of the Baltic states in Eastern Europe. There are about 1.5 million native speakers of Latvian. Of these, 1.38 million live in Latvia, while the rest live in places such as the USA, Russia, Australia, Canada, the United Kingdom, Germany, Lithuania, Estonia, and Sweden (Valoda, 2025). When looking at the second and third generation communities abroad, in many cases, Latvian can be considered as an immigrant heritage language, understood as “a socio-politically minority language acquired as the first or one of the first languages in a bilingual or multilingual context” (Montrul, 2023, p. 399). Latvian as a heritage language (hereafter LAT-HL) is taught in about 100 Latvian diaspora weekend schools—hosted by Latvian communities around the world (Valoda, 2024).

Research about LAT-HL began after the collapse of the Soviet Union and the restoration of Latvia’s independence in 1999. A noticeable increase in research has occurred over the last decade, focusing on family language policy, parental and child agency at home, and the socio-emotional dimension of LAT-HL maintenance in diaspora or, conversely, reimmigration (e.g., Grosa and King, 2023; Martena, 2023; Martena and Burr, 2025). LAT-HL learning has been examined through interviews with and questionnaires of teachers and parents, revealing their perspectives on the role of Latvian diaspora schools in preserving LAT-HL in different social contexts, students’ motivation for learning Latvian, their identities and proficiency in Latvian (e.g., Mieriņa, 2015; Grosa, 2021; Mieriņa et al., 2021). However, the real learning process in the LAT-HL classroom—and students’ thoughts about their learning experiences and linguistic performances in and outside of the classroom—have not been explored to date. Considering this gap in LAT-HL research, this article focuses on the perspective of LAT-HL learners and their agency—“the capability to intentionally and somewhat proactively personalize and otherwise enrich both what is to be learned and the conditions and circumstances under which it is to be learned” (Reeve and Tseng, 2011: 258). To do this, I will use data obtained in four Latvian diaspora schools (one school each in Europe, the US, Canada, and Australia) during an 1-year project (Burr, 2025a): student electronic questionnaires with student reports on the activities experienced in LAT-HL lessons and their role in organizing and implementing the learning process, interviews with students and Latvian-language teachers, texts created by students, and Latvian-language lesson observation checklists. Since the project dealt with the study of texts and literacy improving strategies in the Latvian-language classrooms in Latvia and Latvian diaspora, the LAT-HL learner agency is seen in relation to the development of students’ Latvian proficiency through literacy practices—various learning activities with texts (i.e., reading texts, creating texts, observing/utilizing texts posted on the wall, etc.). And the article aims to capture the manifestations of LAT-HL learner agency when their literacy being developed in the LAT-HL classrooms. Such a perspective is novel for understanding agency in HL classroom settings.

Considering the limited length of the article, the study’s theoretical, methodological, and results parts are presented in a compressed form, highlighting the most important concepts, research steps, and study outcomes. Thus, the theoretical part (Section 2) introduces two main concepts—learner agency and literacy. The methodological part (Section 3) briefly describes both the above-mentioned project and the research approach and data used in this article. The result part (Section 4) presents two components of language learning—language management and learning organization —which enable learner agency in LAT-HL lessons. In the end, I summarize the main conclusions and show directions for future research related to the study of small heritage languages in this thematic scope.

## 2 Theoretical foundation

### 2.1 Learner agency

According to English Language Teaching Expert Panel on learner agency of the Oxford University, “*learner agency* refers to the feeling of ownership and sense of control that learners have when they believe themselves to be active authors of their learning experience rather than passive recipients. When students believe their actions can make a difference, they become more confident, engaged, and effective learners. Agency is not fixed; it is constituted in relationships with others. Thus, the enactment of learner agency may be facilitated or constrained depending on the conditions of the local context” (Larsen-Freeman et al., 2021: 13). Often agency is tied to identity (e.g., Park, 2011), sociocultural (e.g., Ahearn, 2001), and relational (e.g., Edwards, 2005) frameworks. Regardless of the research lens, scholars state that high learner agency includes such classroom practices as

1. students’ generation of their learning content
2. open-ended activities
3. students’ questions to teacher and each other, seeking find answers together
4. students’ teaching other students
5. students’ reflections on lessons, etc. (Larsen-Freeman et al., 2021: 8–12).

As we can see, learner agency manifests in acting more than being acted upon and making responsible decisions and choices instead of accepting those determined by others (OECD, 2019: 4). By doing that learners develop their identity, purposeful learning habits, and a sense of belonging (ibid, 5).

### 2.2 Literacy

Literacy is understood as knowledge, understanding, and the ability to effectively operate with texts. Literacy studies (e.g., Lemov et al., 2016; Steiner and Magee, 2019; Graff, 2022; Mills, 2016) show that from 6th grade to 12th grade, basic literacy is acquired in most parts of the world and crucial proclivities arise in learners such as reading to learn instead of learning to read, passion/apathy/disgust for text reading, and advanced literacy. The multiliteracies approach emphasizes twofold meaning of “*multi-*”: multiple textual practices and multimodality (Cope and Kalantzis, 2015, p. 3). HL research most often talks about *biliteracy* (literacy in two or more languages) among students, with often asymmetrical literacy capabilities in different languages, i.e., children and adolescents having better developed speaking skills but weaker writing skills in their HL (Grose-Hodge, 2024; Hornberger, 2003; Warren, 2017).

The Pedagogy of Multiliteracies proposes a universal learning model with four interrelated knowledge processes: experiencing, conceptualizing, analyzing, and applying (Cope and Kalantzis, 2015, pp. 1–36). It supports learning activities that

1. combine students’ real-life experiences, knowledge, skills, and opinions with new knowledge, experiences, and skills
2. focus on self-directed learning by identifying, naming, categorizing, describing, and concluding about the main concepts, significant elements, models, or texts
3. encourage exposing, evaluating, and interpreting interests and purposes expressed by individuals or social groups in the communication processes, and the possible impact of these interests or purposes on a person, social group, or public opinion
4. focus on putting meanings, knowledge, and understandings to work effectively in the complex diversity of real-world situations (Cope and Kalantzis, 2015, 2020).

Undeniably, texts are essential learning inputs and outputs, which is why learners’ textual practices in the classroom are one of the main prisms for exploring learner agency. And we can see that scholars of both theoretical frameworks emphasize the active involvement of learners in the learning (including their own content creation and reflections on self-directed learning experiences) as an essential and long-term effective experience for them.

## 3 Materials and methods

### 3.1 Description of the postdoctoral project

The article is based on the 1-year project “Development of a Comprehensive Methodology for the In-Depth Study of Students’ Textual Experiences and Literacy Capabilities in the Language Classroom” (2024-2025). During this project, I developed seven research instruments (including a language-classroom observation checklist and an online questionnaire for students about their beliefs of and attitudes toward texts in language classes and out-of-class) for ethnographic linguo-didactics research in the language classroom (Burr, 2025a). All research instruments were tested in eight schools—four schools in Latvia and four schools in the Latvian diaspora—to learn how the proposed methodology in different sociolinguistic and educational environments works in practice. The data and findings included in this article apply to the four Latvian diaspora schools only. They were chosen considering the emigration wave of Latvians during World War II, the number of Latvian schools on each continent and country in the world (Valoda, 2024), and inclusion of countries/schools in previous research by me.^1^ Each school was visited once at the beginning or middle of the school year in early 2025 to test the method and gather data. The study included the Latvian-language classes that most closely correspond to Latvian 6th, 9th, and 12th grades (11- to 18-year-old students)—grades at the end of which students take national tests in the subject, and the age range of students in which, based on theories, students have already acquired basic literacy and are mastering their advanced literacy [more about project’s ethical considerations, progress, and results see in Burr (2025b)].

### 3.2 Research procedures and data of this study

This study is based on a mixed approach combining qualitative data with quantitative data. The dataset consists of 16 language-classroom observations, interviews with 18 Latvian-HL learners, interviews with 6 Latvian-language teachers, 12 LAT-HL learner online questionnaires, 7 student-created texts, and 77 written texts displayed in the classrooms [see samples of all research instruments and their description in Burr (2025a)].

The observation checklists contain written records of all teacher’s and students’ activities throughout lessons (40–60 min), including the teacher’s instructions and students’ questions or comments regarding these instructions, the included texts or tasks to be performed, as well as their mutual conversations about what was happening in the lesson or other topics. All students’ initiatives (e.g., proposed ideas for texts to read, questions, help for classmates), reflections on their learning experiences in the LAT-HL classroom, and comments about the Latvian language, the community, and the diaspora school were first identified, then analyzed and interpreted, considering the above-mentioned learning activities associated with high learner agency (see section “2.1 Learner agency”) and the learning activities defined in the multiliteracy pedagogy module, which both enable students’ knowledge processes—experiencing, conceptualizing, analyzing, and applying—and promote student-centered teaching and student voice in the classroom (see section “2.2 Literacy).

The student questionnaire consists of 18 questions. Its central question is—*Evaluate how often these activities occur in the Latvian-language lessons*—with 78 possible student and teacher actions in the classroom. Students were asked to rate the frequency of learning activities occurring during Latvian-language lessons on a Likert scale—*never, rarely, sometimes, often, always*. Twenty-five statements that relate (or may relate) to learner agency or its limitations were selected for this study. These statements, and students’ answers—*often* and *always*—are provided in Table 1. The analysis of this data is based on the descriptive statistical method.

**TABLE 1:** Statements about working on text in the Latvian-language classroom and the number of the answers showing the occurrence frequency of the statements from the students’ perspective (*N* = *12*).

Statement	Often	Always
You can choose text to read, listen to, or watch and then work with it.	5	–
You all talk together about how to get to know text and complete task more effectively.	6	1
You get to know (read, listen, watch) text and talk about it with other students.	3	–
You get to know (read, listen, watch) text and work with it individually.	8	1
You write out the most interesting or important quotes (fragments) from text.	2	–
You discuss the significance of text in Latvian culture and social life.	5	–
Other students are bothering you with reading and working with text.	4	–
The teacher stops your reading and corrects your mistakes.	4	2
You ask the teacher if you don’t understand something in text or in text-related tasks.	6	–
You ask other students if you don’t understand something in text or in text-related tasks.	5	–
You use auxiliary materials to understand text and complete tasks (e.g., dictionaries, Google, Wikipedia, your notebook).	5	–
You share your thoughts about text and its emotional impact on you.	4	1
You can choose text genre for your creative work.	5	–
You can choose text theme for your creative work.	5	–
You can choose whether to write text (e.g., creative work, application) by hand or computer.	3	1
Other students are bothering you with writing.	4	–
You talk to other students about effective writing strategies.	3	–
Your ideas about evaluating the created text are considered.	7	–
You create oral presentation individually.	7	–
You create oral presentation in group.	5	2
The evaluation of oral presentation will consider the text evaluation criteria you suggest.	6	–
You can choose whether to present from your seat or in front of the class.	5	1
Other students are disturbing your presentation.	5	–
The teacher interrupts your oral speech (e.g., presentation or question) and corrects your mistakes.	3	1
You like to create presentations about Latvian literature and culture.	6	–

^1^Sanita Martena and the author have studied family language policy and multilingual language practices at home. Families were associated to Latvian diaspora schools in New Zealand, United States (Seattle and Los Angeles), Germany, Spain, England, and Norway. The study was financially supported by the State Research Program (2020–2024).

The interviews with 18 students after the observed language lessons capture students’ reflections on their own voice and contribution to LAT-HL lessons, which were uncovered through in-depth content analysis. This method was also used in the analysis of the seven student-created texts entrusted to me and of 77 written texts displayed in the LAT-HL classrooms, revealing students’ contribution to the creation of these texts and of their own learning and communication space.

The dataset and its analysis were used to answer the following research question: Do text-learning activities and literacy practices offered by the teacher in LAT-HL lessons promote learner agency, and, if so, how is it manifested?

## 4 Results

The results of the in-depth analysis of the dataset show that the agency of LAT-HL learners manifests in relation to language management in LAT-HL lessons and co-creation of learning process. Both will be addressed in the following subsections.

### 4.1 Students as setters or challengers of language management in the classroom

The use of Latvian as a free choice of students appears in completing individual text-related tasks or in learning Latvian in general. In seven of the LAT-HL lessons (out of 16) observed, students could, with the teachers’ encouragement or without their interference, choose the language to use in the lesson or in some parts of it. For instance, 7th grade students at a Latvian diaspora school in Europe could use English or another language of society while working on group assignment “Travel plan in Latvia.” Eighth-grade students at a Latvian diaspora school in Canada read, discussed, and wrote their answers to questions about a text about WWII in English, clarifying the meaning of individual words and phrases in Latvian. As the teacher admitted in an interview, “If the students can speak Latvian, great. If not, then the work is mainly in English so that everyone understands.” The above examples reveal the freedom of students’ linguistic choice, while at the same time showing the limitations of the function of Latvian in the learning process.

In turn, two examples represent students’ avoidance of using Latvian. The first example comes from a Latvian diaspora school in Canada where 7th grade students regularly conversed in English during word learning games, and the teacher also addressed them mainly in English. The second example also deals with 7th grade students at a Latvian diaspora school in the United States where they spent an average of 45–50 min per 60-min lesson conversing in English on issues (un-)related to the assigned written work in Latvian. Here the teacher addressed them only when the conversations were too loud and interfered with hearing questions addressed to him about the translation of individual words and sentences into Latvian. Similar to the examples given above, here too, students made a linguistic choice that seemed more appropriate and comfortable to them during the LAT-HL lesson.

The LAT-HL lesson observation checklists include two instances where students talk about their own/other’s Latvian-learning process and the use of Latvian in or outside the classroom; see Excerpt 1 and Excerpt 2.

Excerpt 1 (Grade 7b, Latvian-diaspora school in the United States, 30.03.2025, originally in English)
Student 1: *I have a question: Why do we write every time—why can’t we speak in Latvian sometimes, too, instead of writing things?*
Teacher: *Please, speak Latvian*…. *Why it was not considered this option because it is*…*it is, yeah*…. *I mean.*
Student 2: *I have spoken two words.*
Student 3: *I’m so proud of you.*

Excerpt 1 demonstrates Student 1’s dissatisfaction with LAT-HL learning method offered by the teacher, that is, practicing Latvian through writing tasks and talking in English. As can be seen, the first teacher’s response is an invitation to speak Latvian (indirectly showing that it is not forbidden), but his inability to explain the lack of speaking activities in the lessons shows that he was not ready for the challenge of his teaching style and/or he had not thought about a different way of teaching. The fact that Student 1 dares to ask such a question and even challenge the teacher’s professional competence in front of other students indicates both the student’s capability to initiate potential changes in the LAT-HL learning and an atmosphere in the classroom that allows such conversations to take place at all. Perhaps the presence of the researcher emboldened the student to express her dissatisfaction. The excerpt also shows that the lack of speaking practice has resulted in Student 2’s numerically small use of words, which is ironically commented on by Student 3.

Another example is Excerpt 2. This excerpt shows a short conversation between two students during a break between two LAT-HL lessons. Student 1 is a third-generation Latvian in Australia, while Student 2 is a first-generation Latvian. Both speak Latvian fluently. Student 1 expresses confusion that Student 2, in the first LAT-HL lesson of the double lesson, had difficulty reading the assigned reading passage, e.g., he struggled with pronouncing some words and reread individual words several times. The short communication shows that Student 1 had higher expectations of her classmate’s Latvian reading skills than her own because he was born in Latvia, and that Student 2 justifies his lack of reading skills by not reading (i.e., in all known languages).

Excerpt 2 (Latvian-diaspora school in Australia, 22.02.2025, originally in Latvian).
Student 1: *I don’t understand why you can’t read* [in Latvian—the author], *you’re from Latvia.*
Student 2: *Yes, okay, but I don’t read*…. *In general.*

The last example shows that Student 1 did care what the other students’ Latvian language performance is and was trying to find out the reason for the insufficient reading skill, which in this case was a lack of reading practice.

### 4.2 Student involvement in shaping the learning process and environment

Student involvement in shaping the learning process is closely related to the choices given to them. The most obvious example of student opportunity to alter the learning process was four Latvian-language lessons in grade 7 (ages 12 and 13) on the topic “Love and Valentine’s Day” in a diaspora school in Europe. Students were first tasked with calling one of their parents and finding out about their parent’s first date experience, summarizing the answers they received, and then choosing whether to share their parent’s first date experience with the other students or not, submitting their answers only to the teacher. Then students read the poem “While I Loved You” (Juris Kronbergs) in their own chosen place (the classroom, the corridor, or the library) and whether silently or aloud, without disturbing others. After discussing the poem together, students could choose again the place (as before) where to write their own poem, one of three poem line starters, which they had to include in their poem and complete (“while I loved you, you…,” “while I loved myself, you…,” “while you loved me, I…,” respectively) and then read it aloud or handed it to the teacher. Thus, the teacher-led tasks activate student agency through choices of learning location, text reading, writing, and presentation type that best suit each student’s learning style and self-expression.

Student questionnaire data shows that the option of choosing the topic or genre of creative work is a common practice in LAT-HL lessons; five students (out of 12 students who submitted the questionnaire) have indicated that both choices are offered frequently (see Table 1). Although five students indicated that they can frequently choose a text to work with in class, the observed lessons did not include such cases. However, both the LAT-HL-lesson observations and the student questionnaire data show a similar picture in the organization of work with written text—that is, students more often work with text individually. This form of work organization, as shown by the questionnaire data (see Table 1), was indicated by 9 out of 12 students as frequent or always present. In turn, oral presentation creation often occurs both individually and in groups (see Table 1). In two of the observed LAT-HL lessons (one for 7th grade in Europe and the other for 8th grade in the United States) students created presentations; this work was organized as group work, without the students having the opportunity to choose a different form of work.

When students practice their speaking skills by reading aloud or giving oral presentations, the teacher tends to intervene by correcting spelling, grammar, and syntactic constructions. Observations of LAT-HL lessons showed that teachers focused more on spelling when students read aloud the assigned part of the text, paying less or even no attention to errors in students’ speech (e.g., in answers to questions, during presentations). Questionnaire data confirm this; more students indicated that the teacher corrected errors during reading aloud (see Table 1). On average, four respondents indicated that other students frequently interrupt them while they are reading, writing, or presenting a text.

Classroom observations, interviews with students and teachers, and questionnaire data show a similar picture regarding the feeling of being free to ask questions to the teacher or other classmates if something is unclear in the text they are working with in class, or in language exercises or tasks related to this text. A friendly and free communication-oriented atmosphere was emphasized in interviews with students and teachers several times, highlighting the informal learning environment of Latvian diaspora schools. Students from all Latvian diaspora schools admitted that they always can ask and share their opinions and they practically always feel listened to and heard. However, in only three observed LAT-HL lessons (out of 16), students were asked to share their thoughts about text and the emotional impact of the text on them in the lessons. Five questionnaire respondents indicated that text is seen in the context of Latvian culture and society. Six students—or half of the questionnaire respondents—admitted that they enjoy creating oral presentations about Latvian literature and culture.

In turn, both classroom observation data and questionnaire data show that the excerpting of engaging and interesting fragments for students of written text in language-lessons rarely or never occurs. Similarly, both classroom observation data and questionnaire data show that a few students in the LAT-HL-classroom additionally search for supporting information on the Internet or in learning resources.

When paying attention to teacher/student-led conversations about learning methodology, we see two things: first, there is more frequent joint discussion about how to work with written texts and how to complete tasks related to these texts; second, writing is less discussed among students, with only three students indicating that writing strategies are often discussed with others (see Table 1). But, if we look at the evaluation of student work, we see that half of questionnaire respondents indicated that their ideas and opinions are often considered when evaluating their own texts or their work with texts. These data are neither confirmed nor denied by the observed LAT-HL-lessons, as none of them includes student work assessment (the exception was one 7th grade class in the United States, in which the teacher reviewed and loudly announced two grades of the written work (one for errors and one for content) by two students who finished faster).

Finally, the analysis of the linguistic landscapes of Latvian diaspora schools’ classrooms shows that the role of students in the creation of written texts is minimal or non-existent. In the classrooms, written texts include information about books (titles, authors, publishers) on shelves, lesson plans created by teachers, and examples provided by teachers, e.g., individual words, phrases or sentences in Latvian, sometimes with an English translation, on the board. This is different from the classrooms of younger graders, where many children’s drawings, tables and diagrams, and creative works are placed on the walls and doors. Also, in the common areas—hallways, foyers, libraries—more student-made works were visible. This observation of the lack of visual literacy artifacts indicates limited student opportunities for agentic display.

## 5 Conclusion

To achieve the article goal and answer the research question, I analyzed research dataset and created two thematic groups suggesting student agency: language management in the LAT-HL lessons and student involvement in the creation of the learning process and space. In both cases, the manifestation of learner agency is closely related to

(1)    the teaching methodology chosen by the teacher, which students may or may not accept. The striking example was the teacher’s focus on the strengthening of Latvian through written assignments in grade 7b at a Latvian diaspora school in the United States, and student’s objections to such a constant learning practice (see Excerpt 1),
(2)    the roles of the teacher and students in the classroom. For instance, the participation of 7th-grade LAT-HL students in the European Latvian diaspora school in creating the lesson was greater than the influence of students of a secondary grade in the Australian Latvian diaspora school on the course of the lesson, the role of teachers in both these classes also differed—in one case the teacher was more like an equal participant and consultant to the students, in the other case as a controller and checker of the implementation of the content offered in the textbook,
(3)    the freedom of choice of students, which is suggested by the teacher or circumstances. An excellent example is the choice of language(s) of students. In the LAT-HL lessons in 7th-grade of a European Latvian diaspora school and 8th-grade of a Canadian Latvian diaspora school, teachers encouraged students to use all other languages they knew in completing certain tasks. In turn, the learning process led by the 18-year-old teacher allowed for the free use of English in connection with both the assigned written assignment and other non-study-related matters in the LAT-HL lesson of the 7th grade of the US Latvian diaspora school.

Findings support the idea that agency is situationally produced (Larsen-Freeman), not a stable trait. According to the theory of learner agency (Larsen-Freeman et al., 2021: 8–12), observed LAT-HL lessons and student questionaries show following expressions of learner agency: (1) the inclusion of other languages at times in target language lessons, (2) open-ended activities (e.g., the report of a phone conversation with a parent about a first date), (3) developing students’ ability to adapt the language they learn in the classroom to the contexts in which they need to communicate (e.g., the phone conversation with parents), (4) encouragement to ask questions of the teacher and each other, (5) correcting spelling errors but not punishing students about them. However, they were observed in individual Latvian diaspora LAT-HL lessons. Therefore, although we can see that LAT-HL lessons in Latvian diaspora schools are a place that engages students, learner agency manifest sporadically and not always purposefully promoted by teachers. Thus, methodological work with teachers would be necessary in the future, discussing student-centered teaching and self-directed learning techniques (e.g., student-created learning content, including grammar reminders on walls; creating a journal of the interesting quotes from literary texts, encouraging to find additional information on the topic to be learned, group work on investigation Latvian news, etc.) and their long-term benefits.

The limitations of this article mainly relate to the representation of Latvian diaspora schools, the number of LAT-HL lessons observed, inequivalent student age groups, and observer presence influence. However, I hope that the article will encourage researchers of so-called “small” HLs to pay attention to the voice of students, their capability to change the learning process, and the factors influencing this capability.

## Data Availability

The original contributions presented in this study are included in this article/supplementary material, further inquiries can be directed to the corresponding author.
